# Distress, anxiety, and depression in persons with hereditary cancer syndromes: Results from a nationwide cross‐sectional study in Germany

**DOI:** 10.1002/cam4.5999

**Published:** 2023-05-02

**Authors:** Anna Maria Kastner, Josefine Fischer‐Jacobs, Jan Brederecke, Andrea Hahne, Tanja Zimmermann

**Affiliations:** ^1^ Department of Psychosomatic Medicine and Psychotherapy Hannover Medical School Hannover Germany; ^2^ BRCA‐Netzwerk e.V. Bonn Germany

**Keywords:** anxiety, colorectal neoplasms, depression, hereditary, hereditary breast and ovarian cancer syndrome, hereditary nonpolyposis, neoplastic syndromes, psychological distress

## Abstract

**Background:**

Persons with hereditary cancer syndromes (carriers) have a higher risk of developing cancer early. They are confronted with decisions regarding prophylactic surgeries, communication within their families, and childbearing. The present study aims to assess distress, anxiety, and depression in adult carriers and identify risk groups and predictors; clinicians can use to screen for particularly distressed persons.

**Methods:**

*N* = 223 participants (*n =* 200 women, *n =* 23 men) with different hereditary cancer syndromes affected and unaffected by cancer answered questionnaires measuring their distress, anxiety, and depression levels. The sample was compared to the general population using one‐sample *t*‐tests. The *n* = 200 women with (*n =* 111) and without cancer (*n =* 89) were then compared and predictors for increased levels of anxiety and depression were identified using stepwise linear regression analyses.

**Results:**

66% reported clinical relevant distress, 47% reported clinical relevant anxiety, and 37% reported clinical relevant depression. Compared to the general population, carriers experienced increased distress, anxiety, and depression. Additionally, women with cancer suffered from more depressive symptoms than those without cancer. Past psychotherapy for a mental disorder and high distress were identified as predictors for increased anxiety and depression in female carriers.

**Conclusions:**

The results suggest that the psychosocial consequences of hereditary cancer syndromes are serious. Clinicians could regularly screen carriers regarding anxiety and depression. The *NCCN Distress Thermometer* can be combined with questions about past psychotherapy to identify especially vulnerable persons. Further studies are needed to develop psychosocial interventions.

## 1 INTRODUCTION

Every year, around 492,000 people are newly diagnosed with cancer in Germany.^1^ It is estimated that approximately up to 10% of all cancers have a genetic cause, such as pathogenic variants (PV) and defects in individual genes.^2^, ^3^ There are currently more than 40 known hereditary cancer syndromes, also called *tumor‐predisposition‐syndromes* (TPS).^4^ Most common are *Hereditary Breast and Ovarian Cancer syndrome* (HBOC) and *Hereditary Non‐Polyposis Colon Cancer syndrome* (HNPCC).^5^ Compared to the general population, PV carriers have a significantly increased lifetime risk for developing cancer.^2^, ^3^ Clinical characteristics are early age of onset (age < 60 years), familial accumulation of the syndrome‐specific cancer spectrum, multiple neoplasms at the same time (synchronous cancer), and the reoccurrence of cancer after a short time (metachronous cancer).^3^ Patients with these characteristics should thus be referred to a genetic counselor. A positive test result can lead to a feeling of self‐sufficiency^6^, ^7^, ^8^ as it enables prophylactic surgeries and intensified surveillance for early detection. However, it can also cause psychological stress in carriers, as they need to make difficult decisions regarding prophylactic surgery, family communication, childbearing, insurance, and often have a family history of cancer losses.^9^, ^10^

Most studies to date have only assessed the psychological impact of genetic testing over the period of 1 year after testing and mainly included persons with HBOC or HNPCC. These studies show that while carriers experienced increased levels of distress, anxiety, and/or depression the first weeks after receiving a positive test result, there were no adverse psychological effects after 1 year.^11^, ^12^, ^13^, ^14^ There has been little agreement regarding the mid‐ and long‐term psychological impact to date. Cancer‐unaffected HNPCC carriers reported no increased distress, anxiety, and depression in a three‐year follow‐up study.^15^ Another five‐year follow‐up study, including HBOC carriers, reported higher but no long‐term clinically meaningful psychological burden.^16^ On the contrary, another five‐year follow‐up study with HBOC carriers observed an initial decrease followed by an increase in anxiety and depression from one to five years after testing.^17^

The literature disagrees on the psychosocial consequences of an additional cancer diagnosis. While some studies concluded that cancer had no significant effect on psychological wellbeing,^12^, ^18^, ^19^, ^20^ others had the opposite finding.^11^, ^13^, ^16^, ^21^, ^22^ Although the results are contradictive, the most important risk factors for severe anxiety and depression seem to be a previous cancer diagnosis and high scores before genetic testing,^11^, ^13^ other studies also mentioned familial risk factors, like having lost a family member to hereditary cancer and being a woman with small children.^17^, ^23^

It has not yet been investigated whether and to what extent carriers in Germany suffer from distress, anxiety, and depression. In general, there is a lack of knowledge regarding the psychosocial burden of carriers regardless of the time since testing. To date, it is still unknown which risk groups exist and whether there are feasible predictors that can be used as screening tools in the clinical setting to identify particularly stressed persons. Whereas most studies only examined the psychosocial burden of patients with HBOC or HNPCC, we addressed patients with all kinds of hereditary cancer syndromes.

### 1.1 Study objectives

This study assessed distress, anxiety, and depression that German‐speaking carriers experienced. (1) Due to the unique situation, that many carriers do not have a cancer diagnosis, carriers were compared to the general population in terms of distress, anxiety, and depression. (2) Moreover, female carriers with cancer were compared with those without cancer to identify potential risk groups that are particularly burdened. (3) Another aim was to identify predictors for increased levels of anxiety and depression as age, type of hereditary cancer syndrome, time since diagnosis, relationship status, presence of cancer, the existence of children, existence of a desire to have (more) children, past psychotherapy, and distress measured with the DT.

## 2 MATERIALS AND METHODS

### 2.1 Data sampling

Data in this cross‐sectional multi‐center study were obtained from German‐speaking adults with a range of hereditary cancer syndromes. Due to personnel and financial resources, it was not possible to translate the information material, consent forms, and demographic questions in additional languages and thus to include non‐German‐speaking carriers living in Germany. Recruitment took place from November 2019 to June 2020. Potential participants were identified and approached by geneticists at the national genetic counseling centers of the university hospitals in Germany as well as clinicians at the centers of the German HBOC‐Consortium and HNPCC‐Consortium, who provided them with oral and written information about the study in the form of a flyer. In addition, posters with further information and a link to the online survey were displayed in the waiting rooms of the counseling and consortium centers. Furthermore, patient advocacy groups were involved in recruitment by distributing the flyers to their members and providing information about the study in their email newsletters, on their websites, and social media channels. The involved patient advocacy groups were the German groups for the following syndromes: HBOC, HNPCC, FAP, and MUTYH‐associated‐polyposis, Von Hippel–Lindau syndrome, Gorlin Goltz syndrome, Peutz–Jeghers syndrome, Cowden/Bannayan–Riley–Ruvalcaba/Lhermitte–Duclos syndrome and multiple endocrine neoplasia type. Participants were not compensated for participation. Inclusion criteria consisted of being of legal age, absence of (prior) severe mental illness (participants were asked if they had ever been diagnosed with a mental health disorder for which they have received psychotherapy), and presence of a diagnosis of hereditary cancer syndrome. The online survey was automatically terminated for participants who did not meet the inclusion criteria.

### 2.2 Measures

#### 2.2.1 Distress

Distress was measured using the German version of the *NCCN Distress Thermometer* (DT).^24^ In 1999, the National Comprehensive Cancer Network recommended a routine distress screening in cancer patients. The DT was developed as a simple tool to effectively screen for symptoms of distress. The DT is a tool with well‐established validity and brevity that is available in multiple languages and easy for the provider to interpret, and patients find the DT easy to use. Moreover, the DT is being studied in other patient populations.^25^ Participants rated their subjectively experienced distress in the last week on a single 11‐point scale ranging from 0 (“*No distress”*) to 10 (“*Extreme distress*”). While for a long time a cutoff score of ≥5 was used internationally, the most recent version of the NCCN practice guidelines for distress and a meta‐analysis including 42 studies recommended a cutoff score of ≥4 for clinically relevant distress.^26^, ^27^ Therefore, we used a cutoff of ≥4 for identifying patients with clinically relevant distress and who might be in need of support.

The DT can be supplemented with a problem list that was not used in the present study.

#### 2.2.2 Anxiety

Anxiety levels were measured using the German version of the *Generalized Anxiety Disorder Scale‐7* (GAD‐7). The GAD‐7 is a self‐assessment questionnaire that was developed to diagnose, screen, and measure the severity of generalized anxiety disorder in clinical routine and research. The questionnaire consists of 7 items that are answered regarding the last two weeks and evaluated on a scale from 0 (“*Not at all*”) to 3 (“*Nearly every day*”). The total score is calculated by the addition of the item scores. The total score ranges from 0 to 21 and can be categorized to indicate the severity of the symptoms: Minimal (0–4), mild (5–9), moderate (10–14), and severe (15–20). We used a cutoff of ≥7 for identifying patients with a possible generalized anxiety disorder, as this threshold has the best balance between sensitivity and specificity.^28^ The GAD‐7's internal consistency in the present study was excellent, with *α* = 0.90.

#### 2.2.3 Depression

Depression was assessed with the German version of the *Patient Health Questionnaire*‐9 (PHQ‐9). Participants reported if and to what extent they experienced symptoms of depression in the last two weeks. Answers ranged from 0 (not at all) to 3 (nearly every day). The addition of the item responses accounts for the total score that ranges from 0 to 27. The total score relates to different depression severity groups: None (0–4), mild (5–9), moderate (10–14), moderately severe (15–19), and severe (20–27). We used a cutoff of ≥10 to diagnose clinically relevant depression.^29^ The PHQ‐9's internal consistency in the present sample was excellent, with *α* = 0.87.

### 2.3 Statistical analysis

Differences between the total sample and the general population regarding anxiety and depressive symptoms were tested using one‐sample *t*‐tests. Group differences between women with and without cancer were assessed with linear models that were used to control for the differences across demographics between the groups. To determine risk factors for increased levels of anxiety and depression in women with hereditary cancer syndromes, mixed stepwise logistic regression analyses that combine both forward and backward regressions were used. All statistical analyses were performed in *R*,^30^ and all tests were based on a significance level of 0.05 (if not stated otherwise). Based on a priori power analysis, a total of 98 participants should initially be included in the study to achieve a power of 80% with a medium effect size.

## 3 RESULTS

### 3.1 Participants

The online battery of self‐report measures was initially started by 761 participants, and *N* = 223 completed the whole set of questionnaires resulting in a response rate of 29.83%. Of these, the majority (~80%) had either HBOC (64.57%) or HNPCC (16.59%). The overall sample had an average age of 44.43 years (SD = 11.52, range: 21–76) while the *n* = 200 women (89.69%) were on average 43.74 (SD = 11.28, range: 21–76) years old and the *n* = 23 men (10.31%) were 50.39 years old on average (SD = 12.19, range: 26–70). In the overall sample, the average time since diagnosis of the hereditary cancer syndrome was 53.97 (SD = 61.89, range: 0.23–308) months. While women had an average of 51.43 (SD = 59.56, range: 0.23–308) months, men had an average of 82.65 (SD = 80.60, range: 0.77–231) months. Table 1 displays more detailed information on the sample's demographics and sex comparisons.

**TABLE 1:** Demographic characteristics of the sample by sex.

	Total	Women	Men	*p* ^a^
	(*N* = 223)	(*n* = 200, 89.69%)	(*n* = 23, 10.31%)	
Hereditary cancer syndromes (*n*, %^b^)				
HBOC	144 (64.57)	142 (71.00)	2 (8.70)	<0.001^***^ ^,^ ^c^
HNPCC	37 (16.59)	27 (13.50)	10 (43.48)	
FAP	20 (8.97))	12 (6.00)	8 (34.78)	
Other	22 (9.87)	19 (9.50)	3 (13.04)	
Cancer (*n*, %^b^)				
Yes^e^	118 (52.91)	111 (55.50)	7 (30.43)	0.027^*^ ^,^ ^c^
No	105 (47.09)	89 (44.50)	16 (69.57)	
Months since hereditary cancer syndrome diagnosis (M, SD)	53.97 (61.89)	51.43 (59.56)	82.65 (80.60)	0.137^d^
Age in years (M, SD)	44.43 (11.52)	43.74 (11.28)	50.39 (12.19)	0.019^*^ ^,^ ^d^
Education (*n*, %^b^)				
≤9 years of school	10 (4.48)	8 (4.00)	2 (8.70)	0.123^c^
10 years of school	33 (14.80)	31 (15.50)	2 (8.70)	
>10 years of school	174 (78.03)	157 (78.50)	17 (73.91)	
Other	6 (2.69)	4 (2.00)	2 (8.70)	
Employment status (*n*, %^b^)				
Employed	141 (63.23)	127 (63.50)	14 (60.87)	0.943^c^
Unemployed	64 (28.70)	57 (28.50)	7 (30.43)	
Other	18 (8.07)	16 (8.00)	2 (8.70)	
Children (*n*, %^b^)				
Yes	147 (65.92)	129 (64.50)	18 (78.26)	0.247^c^
Underage (*n*, %^b^ of Yes)	82 (56.55)	73 (57.03)	9 (52.94)	
No	76 (34.08)	71 (35.50)	5 (21.74)	
Past psychotherapy (*n*, %^b^)	108 (48.43)	97 (48.50)	11 (47.83)	1^c^
Relationship status (*n*, %^b^)				
Single	34 (15.25)	34 (17.00)	0 (0.00)	0.051^c^
Married/in a relationship	173 (77.58)	152 (76.00)	21 (91.30)	
Divorced	16 (7.17)	14 (7.00)	2 (8.70)	
Distress (DT) (M, SD)	5.03 (2.68)	5.15 (2.67)	4.00 (2.61)	0.056^d^
Above cutoff (*n*, %^b^)	146 (65.47)	135 (67.5)	11 (47.83)	
Anxiety (GAD‐7) (M, SD)	7.1 (5.12)	7.30 (5.16)	5.39 (4.43)	0.065^d^
Minimal (*n*, %^b^)	85 (38.12)	75 (37.50)	10 (43.48)	
Mild (*n*, %^b^)	69 (30.94)	61 (30.50)	8 (34.78)	
Moderate (*n*, %^b^)	44 (19.73)	40 (20.00)	4 (17.39)	
Severe (*n*, %^b^)	25 (11.21)	24 (12.00)	1 (4.35)	
Depression (PHQ‐9) (M, SD)	7.95 (5.52)	8.24 (5.53)	5.39 (4.89)	0.014^*^ ^,^ ^d^
None (*n*, %^b^)	68 (30.49)	56 (28.00)	12 (52.17)	
Mild (*n*, %^b^)	73 (32.74)	68 (34.00)	5 (21.74)	
Moderate (*n*, %^b^)	51 (22.87)	45 (22.50)	6 (26.09)	
Moderately severe (*n*, %^b^)	25 (11.21)	25 (12.50)	0 (0.00)	
Severe (*n*, %^b^)	6 (2.69)	6 (3.00)	0 (0.00)	

*Abbreviations*: DT, NCCN distress thermometer (cutoff ≥4 for significant distress); FAP, familial adenomatous polyposis; GAD‐7, Generalized Anxiety Disorder Scale‐7 (cutoff ≥7 significant anxiety); HBOC, hereditary breast and ovarian cancer; HNPCC, hereditary non polyposis colon cancer; PHQ‐9, patient health questionnaire‐9 (cutoff ≥10 significant depression).

^*^ *p* < 0.05

*** *p* < 0.001.

^a^ Of the gender comparison.

^b^ All percentages were calculated using valid cases only and sums of more than 100% are due to rounding to two decimal places.

^c^ Fisher's test.

^d^ Welch‐corrected *t*‐test.

^e^ More than one diagnosis was possible, resulting in 68.30% breast and gynecological cancer, 19.70% visceral‐oncological cancer, 1.40% prostate cancer, and 10.60% other cancers in those with cancer.

As the subsample of men was small, only women with and without cancer were compared against each other. The women with (*n* = 111) and without cancer (*n* = 89) differed across a range of demographics, including the distribution of hereditary cancer syndromes (*p* = 0.029), age (*p* < 0.001), employment status (*p* < 0.001), having children (*p* = 0.003), and having undergone psychotherapy in the past (*p* < 0.001; see Table 2).

**TABLE 2:** Demographic characteristics of female participants with and without cancer.

	With cancer (*n* = 111, 55.50%)	Without cancer (*n* = 89, 44.50%)	*p* ^a^
Hereditary cancer syndromes (*n*, %^b^)			
HBOC	76 (68.47)	66 (74.16)	0.029^*^ ^,^ ^c^
HNPCC	20 (18.02)	7 (7.87)	
FAP	3 (2.70)	9 (10.11)	
Other	12 (10.81)	7 (7.87)	
Months since hereditary cancer syndrome diagnosis (M, SD)	54.43 (60.60)	47.65 (58.36)	0.433^d^
Age in years (M, SD)	47.23 (10.28)	39.39 (11.00)	<0.001^***^ ^,^ ^d^
Education (*n*, %^b^)			
≤9 years of school	3 (2.70)	5 (5.62)	0.228^c^
10 years of school	21 (18.92)	10 (11.24)	
>10 years of school	86 (77.48)	71 (79.78)	
Other	1 (0.90)	3 (3.37)	
Employment status (*n*, %^b^)			
Employed	55 (49.55)	72 (80.90)	<0.001^***^, ^c^
Unemployed	45 (40.54)	12 (13.48)	
Other	11 (9.91)	5 (5.62)	
Children (*n*, %^b^)			
Yes	82 (73.87)	42 (47.19)	0.003^**^ ^,^ ^c^
Underage (*n*, %^b^ of Yes)	40 (49.38)	33 (44.00)	
No	29 (26.13)	47 (52.81)	
Past psychotherapy (*n*, %^b^)	67 (60.36)	30 (33.71)	<0.001^***^ ^,^ ^d^
Relationship status (*n*, %^b^)			
Single	13 (11.71)	21 (23.60)	0.084^c^
Married/in a relationship	90 (81.08)	62 (69.66)	
Divorced	8 (7.21)	6 (6.74)	

*Abbreviations*: FAP, familial adenomatous polyposis; HBOC, hereditary breast and ovarian cancer; HNPCC, hereditary non polyposis colon cancer.

* *p* < 0.05

** *p* < 0.01

*** *p* < 0.001.

^a^ Of the group comparison.

^b^ All percentages were calculated using valid cases only, and sums of more than 100% are due to rounding to two decimal places.

^c^ Fisher's test.

^d^ *t*‐test.

### 3.2 Distress, anxiety, and depression in carriers vs. the general population

Carriers had a mean distress score of M = 5.03 (SD = 2.68). As defined by a cutoff score of ≥4 on the DT, the prevalence of clinically relevant psychological distress was 65.47% (*n* = 146) in carriers. One sample *t*‐tests were used to compare the mean scores of carriers and a German general population sample (M = 3.15).^31^ There was a significant difference in the mean distress scores (*t* = 10.469, df = 222, *p* < 0.001, Δ = 1.88) between the two groups, indicating that carriers experience more distress than the general population.

The average anxiety score in the present sample was M = 7.10 (SD = 5.12), with nearly half of the carriers (46.64%, *n* = 104) experiencing anxiety symptoms on a clinically significant level (GAD‐7 ≥ 7). Carriers experienced significantly higher anxiety symptoms than a sample of the general German population (M = 2.97)^32^ (*t* = 12.048, df = 222, *p* < 0.001, Δ = 4.13).

The mean depression score for carriers was M = 7.95 (SD = 5.52). This result was also significantly higher than the mean depression score in a general German population sample (M = 3.6)^33^ (*t* = 11.753, df = 222, *p* < 0.001, Δ = 4.35). Moreover, just over one‐third of carriers (36.77%, *n* = 82) reported clinically significant levels of depression, as defined by the cutoff score of ≥10 in PHQ‐9 scores.

### 3.3 Anxiety and depression in women with cancer vs. women without cancer

As the number of male participants was comparatively low and female and male participants differed across several variables, men were excluded from the following analyses. Additionally, as the two groups of women with and without cancer differed across various demographics (see Table 2), linear models for anxiety and depression were used to examine the association with cancer while controlling for the demographic differences in the groups. A linear regression model with the GAD‐7 score as the outcome variable that controlled for all the available demographics that discriminated between women with and without cancer indicated no significant difference in anxiety scores between the groups, *F*(9,190) = 2.356, *p* < 0.015, adjusted‐*R* ^2^ = 0.06 (see Table 3). However, being younger and a past psychotherapy was associated with higher anxiety scores.

**TABLE 3:** Results of the linear regression analysis for anxiety in female participants.

	Estimate	SE	95% CI		*p*
			LL	UL	
Intercept	10.24	2.11	6.09	14.40	<0.001^***^
Cancer	0.70	0.85	−0.97	2.37	0.411
Disposition syndrome: HBOC	0.64	1.53	−2.38	3.65	0.678
Disposition syndrome: HNPCC	1.84	1.79	−1.68	5.36	0.304
Disposition syndrome: Other	2.71	1.89	−1.02	6.44	0.153
Age in years	−0.09	0.04	−0.16	−0.02	0.015^*^
Employment: Other	−0.58	1.35	−3.25	2.08	0.667
Employment: Unemployed	0.44	0.88	−1.29	2.17	0.618
Children: Yes	0.92	0.78	−0.63	2.46	0.243
Past Psychotherapy: None	−2.26	0.75	−3.74	−0.78	0.003^**^

*Abbreviations*: CI, confidence interval; HBOC, hereditary breast and ovarian cancer; HNPCC, hereditary non polyposis colon cancer; LL, lower limit; *p*, *p*‐value; SE, standard error; UL, upper limit.

* *p* < 0.05

** *p* < 0.01

*** *p* < 0.001.

Another linear regression model with the PHQ‐9 score as the outcome variable that also included all the available demographics that discriminated between the women with and without cancer showed a significant difference in depression scores between women with and without cancer when the demographic differences were controlled, *F*(9,190) = 4.417, *p* < 0.001, adjusted‐*R* ^2^ = 0.13 (see Table 4). Being unemployed and a past psychotherapy was also associated with higher depression scores.

**TABLE 4:** Results of the linear‐regression analysis for depression in female participants.

	Estimate	SE	95% CI		*p*
			LL	UL	
Intercept	10.53	2.16	6.27	14.80	<0.001^***^
Cancer	2.04	0.87	0.33	3.76	0.020^*^
Disposition syndrome: HBOC	−1.18	1.57	−4.27	1.92	0.453
Disposition syndrome: HNPCC	−0.42	1.83	−4.04	3.19	0.818
Disposition syndrome: Other	1.83	1.94	−2.00	5.65	0.347
Age in years	−0.06	0.04	−0.13	0.02	0.124
Employment: Other	1.28	1.39	−1.45	4.02	0.357
Employment: Unemployed	2.00	0.90	0.22	3.77	0.028^*^
Children: Yes	0.43	0.80	−1.15	2.02	0.592
Past psychotherapy: None	−2.32	0.77	−3.84	−0.80	0.003^**^

*Abbreviations*: CI, confidence interval; HBOC, hereditary breast and ovarian cancer; HNPCC, hereditary non polyposis colon cancer; LL, lower limit; *p*, *p*‐value; SE, standard error; UL, upper limit.

* *p* < 0.05

** *p* < 0.01

*** *p* < 0.001.

### 3.4 Risk factors for increased levels of anxiety and depression

To assess possible risk factors for increased anxiety (above the GAD‐7 cutoff) and depression (above the PHQ‐9 cutoff), stepwise logistic regression analyses were performed for each of the given outcomes. All regressions were done using the same pool of possible predictors that are expected to be at hand for clinicians: Age, type of hereditary cancer syndrome, time since diagnosis, relationship status, presence of cancer, the existence of children, existence of a desire to have (more) children, past psychotherapy, and distress measured with the DT.

A stepwise logistic regression analysis with the dichotomized anxiety variable (above vs. below the GAD‐7 cutoff) as the outcome converged into a model that differed in a statistically significant way from a null model, *χ* ^2^(6) = 46.796, *p* < 0.001, McFadden‐*R* ^2^ = 0.18. This model included the (non‐)existence of a relationship, the kind of hereditary cancer syndrome, the (non‐)existence of a past psychotherapy, and the (non‐)existence of high levels of distress measured with the DT as predictors. Of these, only the (non‐)existence of a past psychotherapy (Wald's *χ* ^2^ = 4.858, *p* = 0.028) and the (non‐)existence of high levels of distress measured with the DT (Wald's *χ* ^2^ = 25.332, *p* < 0.001) were of statistical significance. Additionally, this model correctly predicted 66.67% of the anxiety values in the present sample. For reasons of parsimony, a model that only included the statistically significant predictors was then tested against the initial model. The result of this likelihood‐ratio test indicated no statistically significant difference of the models, *χ* ^2^(4) = 7.197, *p* = 0.126. When this reduced model was used to predict increased levels of anxiety, it correctly identified 68.23% of the sample, slightly improving the prediction rate. It was thus decided to keep the smaller, more parsimonious model as the final model (see Table 5).

**TABLE 5:** Results of the final logistic regression model for dichotomized anxiety in female participants.

	OR	95% CI		Wald's *χ* ^2^	df	*p*
		LL	UL			
Intercept	0.33	0.15	0.67	–	–	0.003^**^
Past psychotherapy: None	0.47	0.25	0.88	5.46	1	0.019^*^
Distress: Above threshold	7.03	3.42	15.5	25.92	1	<0.001^***^

*Note*: Likelihood‐ratio test against null‐model: *χ* ^2^(2) = 39.599, *p* < 0.001, McFadden‐*R* ^2^ = 0.15.

*Abbreviations*: CI, confidence interval; Df, degrees of freedom; LL, lower limit; OR, odds ratio; *p*, *p*‐value; UL, upper limit.

* *p* < 0.05

** *p* < 0.01

*** *p* < 0.001.

The stepwise logistic regression model for the dichotomized depression variable (above vs. below the PHQ‐9 cutoff) also converged into a model that differed in a statistically significant way from a null model, *χ* ^2^(7) = 53.865, *p* < 0.001, McFadden‐*R* ^2^ = 0.21. This model included the (non‐)existence of a relationship, (non‐)existence of cancer, the kind of hereditary cancer syndrome, the (non‐)existence of past psychotherapy, and the (non‐)existence of high levels of distress measured with the DT as predictors. Again, only the (non‐)existence of past psychotherapy (Wald's *χ* ^2^ = 5.58, *p* = 0.018.) and the (non‐)existence of high levels of distress measured with the DT (Wald's *χ* ^2^ = 19.950, *p* < 0.001) were of statistical significance. This model predicted 71.88% of the dichotomous depression values correctly. Analog to the anxiety model, a smaller, more parsimonious model was tested against the initial model. This likelihood‐ratio test indicated a significant difference in the model fit, *χ* ^2^(5) = 11.271, *p* = 0.046. Nonetheless, the smaller model still correctly predicted 70.31% of the sample's dichotomous depression scores. As this was not interpreted as a clinically relevant decrease in accuracy, the smaller model (see Table 6) was preferred as the final model.

**TABLE 6:** Results of the final logistic regression model for dichotomized depression in female participants.

	OR	95% CI		Wald's *χ* ^2^	Df	*p*
		LL	UL			
Intercept	0.18	0.07	0.4	–	–	<0.001^***^
Past psychotherapy: None	0.39	0.2	0.74	8.08	1	0.004^**^
Distress: Above threshold	9.18	3.91	25.4	22.24	1	<0.001^***^

*Note*: Likelihood‐ratio test against null‐model: *χ* ^2^(2) = 42.595, *p* < 0.001, McFadden‐*R* ^2^ = 0.17.

*Abbreviations*: CI, confidence interval; Df, degrees of freedom; LL, lower limit; OR, odds ratio; *p*, *p*‐value; UL, upper limit.

** *p* < 0.01

*** *p* < 0.001.

## 4 DISCUSSION

This study examined German‐speaking adults with different hereditary cancer syndromes with and without cancer regarding distress, anxiety, and depression. The aim was to identify risk groups and predictors for high levels of anxiety and depression that physicians can use to screen for particularly burdened patients.

Around 66% of carriers reported clinically relevant distress and 47% reported anxiety levels indicating generalized anxiety disorder. Another 37% of carriers experienced depressive symptoms corresponding to clinical depression. The sample experienced more distress, anxiety, and depression than the general population.

This is consistent with studies comparing anxiety and depression among cancer patients to the general population.^34^ To our knowledge, there are no other published studies that compare distress, anxiety, and depression levels of carriers to the general population.

Looking more closely at female carriers, it was found that women with cancer experienced more depression than women without cancer. Both subgroups showed no difference in anxiety. The findings are only partly consistent with studies including HBOC carriers, which showed that 3 and 6 months after genetic testing, levels of anxiety and depression were higher in cancer‐affected carriers than in those without cancer.^11^, ^13^ Furthermore, in female carriers, a past psychotherapy was associated with higher anxiety and depression scores, as was young age for anxiety and unemployment for depression. Previous studies also reported that younger women experienced more (cancer‐related) anxiety.^35^, ^36^

The question remains, how can clinicians recognize particularly burdened carriers? Which screening tools are suitable in practice and in which situations can screening be implemented? Assuming that a clinician would have the following possible predictors at hand during a routine appointment: age, type of hereditary cancer syndrome, time since diagnosis, relationship status, presence of cancer, the existence of children, existence of a desire to have (more) children, past psychotherapy and distress measured with the DT. Of these, only the distress score and a history of psychotherapy were significant predictors of high anxiety and depression in female carriers in the present study.

The findings indicate that the DT is an effective screening tool. Since it consists of only one question, it is also quick and easy to apply in the clinical setting. Additionally, asking about past psychotherapy for mental health disorders can indicate particularly vulnerable persons. A Canadian research team also developed a 20‐item *Genetic Psychosocial Risk Instrument* to screen at‐risk individuals undergoing genetic testing and determine those that would benefit most from psychosocial support.^37^ However, the focus is on psychosocial problems that arise in the course of genetic testing and the 20 items may not be practical in a clinical setting.

Female carriers with cancer make up a risk group for particularly high levels of depressive symptoms. However, they may have easier access to psychosocial support as part of their psycho‐oncological care. Therefore, the screening of carriers with cancer can take place at treatment and follow‐up appointments, as it is already implemented in many clinics.

Although they do not constitute a risk group, the results show that female carriers without cancer also experience distress, anxiety, and depression to a large extent. Screening is more difficult here, as cancer‐unaffected carriers are rarely seen in the hospital. In addition, they do not have one clinical department as a consistent contact, as different disciplines are involved in the care depending on the hereditary cancer syndrome. This places high demands on interdisciplinary communication and agreements regarding responsibility. Therefore, it is important that both clinicians and physicians in private practices also regularly screen cancer‐unaffected carriers for psychosocial distress. This could become a routine at surveillance and early detection appointments for cancer‐unaffected carriers. Depending on the score, all carriers may be offered a referral to mental health professionals.

### 4.1 Limitations

There is a number of limitations to the study that need to be considered. First, the data are based entirely on self‐report measures and the study design is cross‐sectional; these methods can pose issues that are common in psychological research, namely response bias and common method variance. Since participation in the online survey was not supervised by healthcare professionals, compliance with the inclusion and exclusion criteria relies on the trust of honest responses from participants. Furthermore, the type of healthcare professional who had to diagnose the condition was not specified. This is a limitation because we cannot be sure that only adults, hereditary cancer syndromes carriers and persons without a pre‐existing mental health disorder, for which they have received psychotherapy, participated in the study. Moreover, despite its size, the sample is not representative in terms of the distribution of gender, hereditary cancer syndromes, cancer status, and type which could impact the results regarding differenced in anxiety and depression. The uneven distribution of the different hereditary cancer syndromes is a limitation, although partly explainable by the different incidences of the different hereditary cancer syndromes, which are assumed to be HBOC 1:500 to 1:1000, HNPCC 1:500, FAP 1:33,000.^5^ Further studies are warranted to examine the extent to which HCS type influences anxiety, depression, and distress. In particular, it is a limitation that there were only few male participants and no non‐binary participants. Furthermore, participants were partly recruited through patient advocacy groups. It may be that distressed persons in particular seek support there, and therefore, persons with increased psychosocial distress are more likely to have participated in the study. Since all questionnaires were in German, our study population contains only German‐speaking persons.

## 5 CONCLUSIONS

Carriers experienced more distress, anxiety, and depression than the general population. Around 66% of all carriers reported clinically relevant distress, 47% reported anxiety levels indicating generalized anxiety disorder and 37% experienced depressive symptoms corresponding to clinical depression. Female carriers with cancer experienced more depression than female carriers without cancer. There was no difference in anxiety. A past psychotherapy was associated with higher anxiety and depression scores in female carriers, as was young age for anxiety and unemployment for depression. Distress measured with the DT and a history of psychotherapy were significant predictors of higher anxiety and depression symptoms in female carriers in the present study.

The DT and asking about past psychotherapy for a mental health disorder can be used to screen particularly vulnerable carriers. Regular distress screening at each visit to a medical health professional could be implemented. To ensure this, distress screening could be included in national clinical and local hospital guidelines. Carriers with high levels of distress may be offered further help and referral to a mental health professional. However, it is important that also psychosocial support services adapted to the carrier's situation are developed and implemented. Psycho‐oncological services for carriers affected by cancer are important, as they are particularly burdened. Support services for carriers without cancer or who have survived cancer could be offered, as these two groups are not able to access psycho‐oncological services. In general both services should be made available throughout the country, also in rural areas. This study provided further insight into the psychological burden carries for hereditary cancer syndromes carry. As genetic diagnostic technologies continue to evolve, it is expected that more hereditary cancer syndromes will be discovered and thus more people will be diagnosed in the future. Given the variety of syndromes, that have already been identified, and their diverse organ manifestations, healthcare professionals from almost every medical specialty are involved in the care of carriers. Therefore, it is crucial to spread medical knowledge about hereditary cancer syndromes and to raise awareness of the special psychosocial burdens carriers and their families are facing. Further studies should examine male and non‐binary carriers in particular, as they were underrepresented in our study. In general, further research is needed to accurately assess the needs of carriers and to develop psychosocial care services and interventions in the future.

## AUTHOR CONTRIBUTIONS

**Anna Maria Kastner:** Conceptualization (equal); data curation (equal); formal analysis (equal); funding acquisition (supporting); investigation (lead); methodology (equal); project administration (equal); resources (equal); software (lead); validation (equal); visualization (equal); writing – original draft (lead); writing – review and editing (lead). **Josefine Fischer‐Jacobs:** Conceptualization (equal); formal analysis (equal); funding acquisition (supporting); investigation (lead); methodology (equal); project administration (equal); resources (equal); software (equal); validation (equal); visualization (equal); writing – original draft (supporting); writing – review and editing (supporting). **Jan Brederecke:** Data curation (equal); formal analysis (equal); methodology (equal); software (equal); validation (equal); writing – original draft (supporting); writing – review and editing (supporting). **Andrea Hahne:** Conceptualization (supporting); investigation (supporting); resources (supporting); writing – review and editing (supporting). **Tanja Zimmermann:** Conceptualization (lead); formal analysis (equal); funding acquisition (lead); investigation (supporting); methodology (equal); project administration (supporting); resources (equal); supervision (lead); validation (equal); writing – original draft (equal); writing – review and editing (equal).

## FUNDING STATEMENT

The study was part of the KlinStrucMed Graduation Program, funded by the Else Kröner‐Fresenius‐Stiftung. The funding source had no influence on the design or execution of the study, data analysis or interpretation.

## CONFLICT OF INTEREST STATEMENT

The authors declare that they have no conflict of interest.

## ETHICS APPROVAL STATEMENT

The study and the procedures were approved by the ethical committee of the Hannover Medical School, Germany (Approval No. 8541_BO_K_2019).

## PATIENT CONSENT STATEMENT

All participants were informed in written form before participation and gave written informed consent before taking part in the online survey.

## Data Availability

The datasets generated during and/or analyzed during the current study are available from the corresponding author on reasonable request.
